# Divergent impacts on the gut microbiome and host metabolism induced by traditional Chinese Medicine with Cold or Hot properties in mice

**DOI:** 10.1186/s13020-022-00697-2

**Published:** 2022-12-26

**Authors:** Bingbing Li, Xin Tao, Lili Sheng, Yan Li, Ningning Zheng, Houkai Li

**Affiliations:** 1 School of Pharmacy, Shanghai University of Traditional Chinese Medicine, Shanghai, 201203 China; 2 School of Life Science, Westlake University, Hangzhou, 310000 China

**Keywords:** Gut microbiota, Host metabolism, Hot–Cold theory, Serum metabolomics, Traditional Chinese Medicine (TCM)

## Abstract

**Background:**

Traditional Chinese Medicine (TCM) has been practiced and developed in China over thousands of years under the guidance of a series of complicated traditional theories. Herbs within TCM usually are classified according to their different properties ranging from cold, cool, warm to hot, which are simplified as Cold and Hot properties. TCM with either Cold or Hot properties are used in various formulae designed for the purpose of restoring the balance of patients. Emerging evidence has highlighted that an altered gut microbiota or host metabolism are critically involved in affecting the healing properties of TCM. However, at present the exact influences and crosstalk on the gut microbiota and host metabolism remain poorly understood.

**Methods:**

In the present study, the divergent impacts of six TCMs with either Cold or Hot properties on gut microbiome and host metabolism during short- or long-term intervention in mice were investigated. Six typical TCMs with Hot or Cold properties including *Cinnamomi Cortex* (rougui, RG), *Zingiberis Rhizoma* (ganjiang, GJ), *Aconiti Lateralis Radix Praeparata* (fuzi, FZ), *Rhei Radix et Rhizoma* (dahuang, DH), *Scutellariae Radix* (huangqin, HQ), and *Copitdis Rhizoma* (huanglian, HL) were selected and orally administered to male C57BL/6J mice for a short- or a long-term (7 or 35 days). At the end of experiments, serum and cecal contents were collected for metabolomic and gut microbiome analyses using gas chromatography–tandem mass spectrometry (GC–MS) or 16S ribosomal deoxyribonucleic acid (16S rDNA) sequencing.

**Results:**

The results revealed that the gut microbiome underwent divergent changes both in its composition and functions after short-term intervention with TCM possessing either Cold or Hot properties. Interestingly, the number of changed genus and bacteria pathways was reduced in Hot_LT, but was increased in Cold_LT, especially in the HL group. Increased α diversity and a reduced F/B ratio revealed the changes in Hot_ST, but a reduced Shannon index and increased altered bacteria function was evident in Cold_LT. The serum metabolic profile showed that the influence of TCM on host metabolism was gradually reduced over time. Glycolipid metabolism related pathways were specifically regulated by Hot_ST, but also surprisingly by Cold_LT. Reduced lactic acid in Cold_ST, increased tryptophan concentrations and decreased proline and threonine concentrations in Cold_LT perhaps highlighting the difference between the two natures influence on serum metabolism. These metabolites were closely correlated with altered gut microbiota shown by further correlation analyses.

**Conclusion:**

The results indicated that TCM properties could be, at least partially characterized by an alteration in the gut microbiota and metabolic profile, implying that the divergent responses of gut microbiome and host metabolism are involved in different responses to TCM.

**Graphic Abstract:**

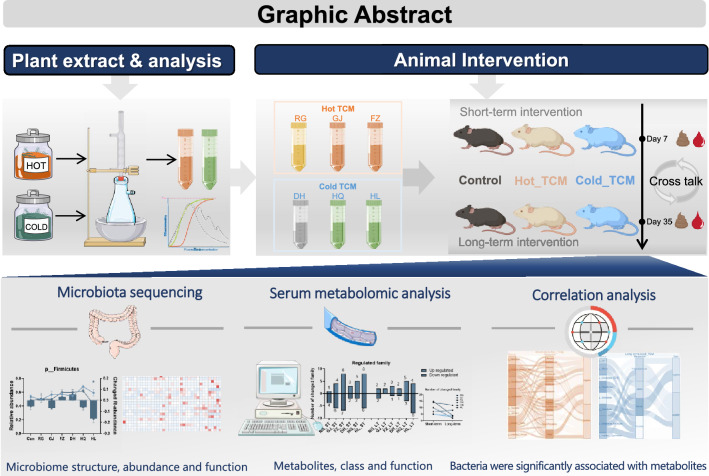

**Supplementary Information:**

The online version contains supplementary material available at 10.1186/s13020-022-00697-2.

## Introduction

Traditional Chinese Medicine (TCM) has been practiced and developed over thousands of years in China. In past decades, there has been an increasing focus on the clinical applications of TCM, as well as fundamental research on the scientific basis of TCM theory worldwide [1, 2]. However, the complexity of TCM theories and numerous chemical components within each herbal remedy is still a great challenge for the modernization of TCM [3]. The TCM nature (Yao Xing in Chinese) including Cold, Cool, Hot and Warm is a type of basic TCM characteristic that was recorded in “Shen Nong’s Materia Medica (Shen Nong Ben Cao Jing)” about 2000 years ago. Although we still know little about the scientific basis of TCM nature, different herbs with either Hot or Cold properties were selectively used in TCM formulae for treating diseases with the opposite Syndrome (Zheng in Chinese) by TCM physicians in the clinic, that is “Treating the Hot Syndrome with Cold nature medicine and treating the Cold Syndrome with Hot nature medicine” [2, 4–6].

In recent years, many studies have been carried out to explore the biological basis of TCM nature by testing the different impacts of Hot or Cold TCM on body temperature regulation [7–9], host energy metabolism [10–12], including Na^+^/K^+^ ATP enzyme activity [13, 14], and so on. Previously, we compared the divergent influences of Hot or Cold TCM on the physiology of mice by given orally Hot or Cold TCM for 7 or 35 days, including *Cinnamomi Cortex* (rougui, RG), *Zingiberis Rhizoma* (ganjiang, GJ), *Aconiti Lateralis Radix Praeparata* (fuzi, FZ), *Rhei Radix et Rhizoma* (dahuang, DH), *Scutellariae Radix* (huangqin, HQ), and *Copitdis Rhizoma* (huanglian, HL). The results indicated that a short-term Hot or Cold TCM intervention exerted a more significant impact on lipid metabolism in the liver or adipose tissue than a long-term intervention [15, 16], suggesting host adaptation to long-term TCM intervention. In addition to a direct influence on host physiology, gut microbiome modulation is the intermediary pathway for TCM actions [17, 18]. Recently, Wu et al. investigated the effects of 12 TCMs with different degrees of Hot or Cold nature on gut microbiota in mice and found that the influences on gut microbiota was in the order: Cold > Cool > Warm > Hot TCM [19]. It is reasonable to assume that Cold TCM usually contained significantly more alkaloids than Hot TCM, which possess anti-bacteria activity [20]. In addition, the interaction between TCM and gut microbiota may give rise to the production of microbial metabolites, as well as the regulation of host metabolism simultaneously [21, 22]. Unfortunately, there is a paucity of studies on TCM nature that have incorporated changes in the gut microbiota and host metabolism at same time.

In the current study, the divergent impacts of Hot or Cold TCM on the gut microbiome and host metabolism in mice were investigated by orally giving aqueous extractions of six TCMs with typical Hot or Cold nature for a short-term (7 days) and a long-term (35 days), interventions based on 16S ribosomal deoxyribonucleic acid (16S rDNA) and untargeted metabolomic approaches. The results revealed that TCM with a Hot or Cold nature exerted unique influences on the microbiome and host metabolism after either the short-term or long-term interventions. The correlation analysis suggested an interplay between the altered gut microbiome and metabolic pathways that were specifically modulated by Hot or Cold TCM.

## Materials and methods

### Plant extract materials

Six TCMs of RG (production batch: 200702), GJ (200415), FZ (200706), DH (200717), HQ (200701), and HL (200820), were purchased from Shanghai KangQiao Chinese Medicine Co. Ltd. (Shanghai, China), and were authenticated by a professor of the Institute of Chinese Medicine, Shanghai University of Traditional Chinese Medicine. A voucher specimen of the individual TCMs was deposited at the School of Pharmacy, Shanghai University of Traditional Chinese Medicine. Approximately 100 g raw material of each TCM was soaked in ultrapure water for 4 h before extraction. For GJ, HL and HQ, they were boiled violently for 15 min and then kept slightly boiling for a further 45 min. A reflux extraction device was used in the whole process [23–25]. RG and DH were boiled violently for 25 min and then kept slightly boiling for 65 min [26, 27]. FZ was boiled violently for 10 min and then kept slightly boiling for 20 min [28]. Each TCM was extracted twice and the filtrate combined and concentrated at 60 °C under reduced pressure. A shorter extraction time was selected based on the content of volatile components in RG and anthraquinone in DH. A longer extraction time aimed to reduce the toxic components in FZ. These water extracts were then used to analyze the components in the next step.

### Compositional analysis of the aqueous extract

Ultrahigh performance liquid chromatography coupled with quadrupole time flight mass spectrometry (UPLC–QTOF-MS/MS) analysis was performed on an Agilent 1290 UPLC system coupled to Agilent 6545 Q-TOF instrument (Agilent, Santa Clara, CA, USA). The spectrometer was operated in full-scan TOF–MS at m/z 50–1500 and information-dependent acquisition (IDA) MS/MS modes. The specific conditions were: gas temperature (320 °C), Drying Gas (11 L/min), Nebulizer (35 psi), Shealth Gas Temp (350 °C), Shealth Gas Flow (11 L/min), and Collision Energy (40 eV). Chromatographic separation was achieved using an Agilent ZORBAX RRHD Eclipse Plus C18 (3.0 × 100 mm, 1.8 μm) analytical column. The mobile phase consisted of 0.1% formic acid (v/v) in water and acetonitrile. The temperature (°C), detection wavelength (nm), and velocity of flow (mL/min) were 30, 260 and 0.5 for RG, 40, 215 and 0.3 for GJ, 30, 190–400 and 0.3 for FZ, 25, 260 and 0.3 for DH, and 30, 280 and 0.3 for HQ. For HL, HPLC analysis was obtained for stable baseline and coupled to Agilent ZORBAX Extend-C18 (4.6 mm × 250 mm, 5 µm, 25 °C, sample injection volume 2 µL). The mobile phase consisted of acetonitrile and 0.1% trifluoroacetic acid solution. Each TCM was analyzed by a specific gradient elution procedure.

### Animal experiments

Male C57BL/6J 4-week-old mice were provided by the Laboratory Animal Center of Shanghai University of Traditional Chinese Medicine (Shanghai, China). Mice were orally administered a vehicle or respective test extracts once daily for 7 days or 35 days after 1-week adaptive feeding (approval number of ethics protocol: PZSHUTCM200918014). The mouse dose was converted from the maximum clinical dose specified in the Pharmacopoeia of the People’s Republic of China (2020 edition) according to body surface area, that is, 0.65 g/kg for RG, 1.3 g/kg for GJ, 1.95 g/kg for FZ, 1.95 g/kg for DH, 1.3 g/kg for HQ and 0.65 g/kg for HL. All mice were housed in a 12-h light (7 am to 7 pm) and 12-h dark (7 pm to 7 am) cycle, with ad libitum access to water and laboratory chow. The experiments were conducted under the Guidelines for Animal Experiment of Shanghai University of Traditional Chinese Medicine and the protocol was approved by the institutional Animal Ethics Committee.

### 16S rDNA sequencing and untargeted metabolomics

Fecal deoxyribonucleic acid (DNA) was isolated for 16S rDNA sequencing and deproteinized serum was collected for untargeted metabolomics. For 16S rDNA sequencing, fecal DNA was isolated using the Qiagen QIAamp DNA Stool Mini Kit (Qiagen, Dusseldorf, Germany) and Illumina sequencing was carried out based on published methods [29, 30]. Then the V3–V4 region was amplified and sequenced. QIME software 1.9.1 was used to analyze the sequence readings, and PICRUSt2 analysis was used to predict microbial community functions. For untargeted metabolomics, the serum was centrifuged at 4 °C and 3000*g* for 5 min to separate the debris or lipid layer. Internal standard (10 µL) was added to 50 µL of the supernatant, slightly mixed, and then 175 µL of a precooled methanol/chloroform (V/V = 3:1) mixture added. The samples were centrifuged at 14,000*g* and 4 °C for 20 min, and then stood at − 20 °C for 20 min. For 200 µL samples of the supernatant the chloroform was removed then they were and freeze dried. Next, high-purity nitrogen was flushed for protection and placed on the automatic derivation platform Xplore MET for silane derivatization. Ion peaks were determined and denoising baseline was corrected in Xplore MET, then the metabolite peaks were resolved by fast gas chromatography–time of flight mass spectrometry (GC–TOF/MS). All the steps followed methodology previously published [31, 32]. Sequence data associated with this project have been deposited in the NCBI Short Read Archive (PRJNA832599, PRJNA832616) database.

### Statistical analysis

Data shown in this study are expressed as the mean ± SEM unless stated otherwise. Differences between groups in microbiota and functions were evaluated using the Mann–Whitney *U* test. Other comparisons between two groups were conducted using the two-tailed Student’s *t*-test. A *P*-value < 0.05 was considered to be a statistically significant finding.

## Results

### Short-term TCM intervention alters the composition and functions of the gut microbiota

**Fig. 1:**
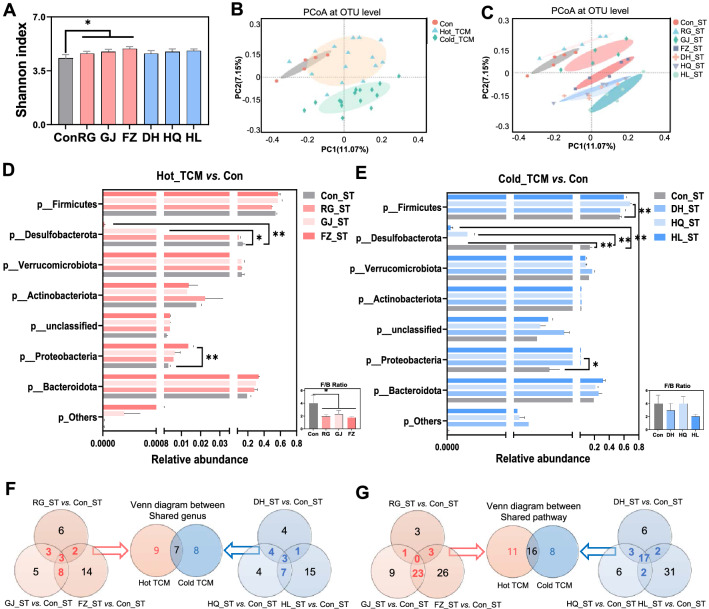
The effect of TCM short-term intervention on gut microbiota composition and function. Male C57BL/6J mice (5-week-old) were given Hot TCM (RG_ST, GJ_ST, FZ_ST) or Cold TCM (DH_ST, HQ_ST, HL_ST) by oral gavage once daily for 7 days. Mice in control group were orally administrated with water (Con_ST). **A** Shannon index. **B**, **C** Principal coordinate analysis based on the Binary-Pearson distance algorithm on OTU level. **D**,** E** Fecal microbiota at phylum level by 16S rDNA sequencing under Mann–Whitney *U* test. **F** Venn diagram between Hot TCM and Cold TCM based on shared genus of each nature. **G** Venn diagram shown the overlap between Hot TCM shared pathway and Cold TCM shared pathway by PICRUSt2 analysis on KEGG pathway level 3 under Mann–Whitney *U* test. N = 5 each group, *P < 0.05, **P < 0.01

To investigate the impact of short-term TCMs with either Cold or Hot properties on the composition and functions of the gut microbiome, C57BL/6J mice were given TCM extracts or vehicle once a day for 7 days. The 16S rDNA sequencing results for fecal bacteria showed an increased richness and evenness of the gut microbiome after a Hot TCM (RG, GJ and FZ) intervention, but not Cold TCM (Fig. 1a). However, the PCoA at the operational taxonomic units (OTU) level showed that Hot TCM treated mice were closer to the Control group than Cold TCM treated mice, suggesting Cold TCM (DH, HQ and HL) exerted more impacts on the structural composition of the gut microbiota than Hot TCM (Fig. 1b, c). Among the Hot TCM treated groups, the relative abundance of Proteobacteria was significantly increased in FZ group, while Desulfobacterota was reduced in both the FZ and GJ groups (Fig. 1d). In contrast, decreased relative abundance of Desulfobacterota and increased Proteobacteria were observed in the 3 groups of Cold TCM, while Firmicutes was only significantly increased in the HQ group (Fig. 1e). Additionally, the Firmicutes-to-Bacteroidetes ratio (F/B ratio) was universally reduced by Hot TCM, but not by Cold TCM. To compare the common or unique characteristics that were altered by Hot or Cold TCM short-term intervention, Venn analysis was performed based on the changed microbiota at the genus level. First, the regulated genus in RG, GJ and FZ were used in the Venn analysis, and 16 genera which were regulated by two and three water extracts simultaneously were considered as Hot TCM regulated genus. Then, 15 genera were observed in Cold TCM in the same way. Next, the above 16 Hot TCM regulated genus and 15 Cold TCM regulated genus were involved for further Venn analysis and 7 genera were found to be significantly altered by both Hot and Cold TCM, while the remaining 9 and 8 genera were uniquely altered by Hot TCM and Cold TCM respectively (Fig. 1f, Additional file 1: Fig. S1A). In the 7 shared genus, only *g__Allobaculum* was reverse regulated, which was increased by Hot_ST and decreased by Cold_ST, the remaining genus was decreased by both natures. Hot TCM intervention specifically increased the abundance of *g__Tuzzerella*, *g__Eubacterium_oxidoreducens_group*, *g__unclassified_c__Clostridia*, and *g__Colidextribacter*, and decreased the abundance of *g__UBA1819*, *g__unclassified_p__Firmicutes*, *g__Enterorhabdus*, *g__Lactobacillus* and *g__Faecalibaculum*. The Cold TCM intervention increased the abundance of *g__Bacteroides* and *g__Bacteroides*, and decreased the abundance of *g__unclassified_f__Erysipelotrichaceae*, *g__Clostridium_sensu_stricto_1*, *g__DNF00809*, *g__Muribaculum*, *g__Candidatus_Saccharimonas* and *g__Odoribacter*.

To reveal the impact on microbial functions after a TCM intervention, PICRUSt2 analysis based on the Kyoto Encyclopedia of Genes and Genomes (KEGG) pathway was performed. At KEGG pathway level 3, Venn analysis between Hot and Cold TCM showed that more changes were induced by Hot TCM on microbial functions (Fig. 1g), 11 pathways were uniquely changed by Hot TCM, and most of them belonged to Metabolism at level 1, including the adipocytokine signaling pathway, tyrosine metabolism, d-arginine and d-ornithine metabolism. While 8 pathways were only changed by Cold TCM and most belonged to environmental information processing at level 1 including the cAMP signaling pathway (Fig. 1g, Additional file 1: Fig. S1B). In addition, the capability of the gut microbiome on aromatic compound degradation and drug metabolism were regulated by both Hot and Cold TCM. In summary, short-term intervention with Hot TCM significantly increased microbiota diversity, while Hot and Cold TCM made significant but different changes to the gut microbiota and its functions.

### The compositional and functional changes of gut microbiota after long-term TCM intervention

**Fig. 2:**
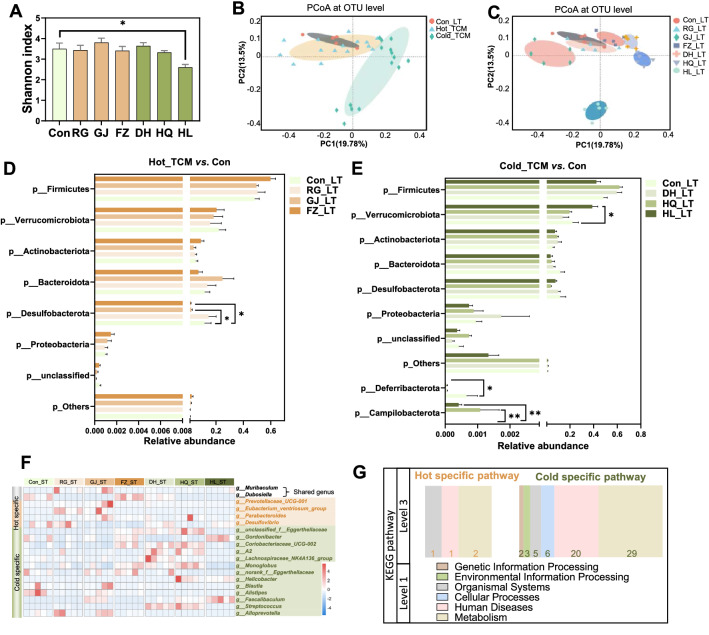
The distinct regulation on gut microbiome by TCM after 35 days intervention. Male C57BL/6J mice (5-week-old) were given Hot TCM (RG_LT, GJ_LT, FZ_LT) or Cold TCM (DH_LT, HQ_LT, HL_LT) by oral gavage once daily for 35 days. Mice in Control group were orally administrated with water (Con_LT). **A** Shannon index. **B**, **C** Principal coordinate analysis based on the Binary-Pearson distance algorithm on OTU level. **D**, **E** Fecal microbiota at phylum level by 16S rDNA sequencing under Mann–Whitney *U* test. **F** Heatmap shown the specific genus of Hot_LT and Cold_LT. **G** The number of regulated pathways in two TCM natures at KEGG pathway level 3. N = 5 each group, *P < 0.05, **P < 0.01

Given that the TCM treatment period is highly variable in the clinic, we intended to explore the impact of long-term TCM intervention on mice during a 35-day treatment period. In contrast to the short-term intervention, only HL treated mice showed a decreased value in the Shannon index (Fig. 2a), suggesting reduced α diversity of the gut microbiota. In line with the short-term intervention (Fig. 1b), PCoA at the OTU level showed that the long-term Cold_TCM group was clearly separated with the Con group alongside PC1, but not the Hot_TCM group (Fig. 2b, c). Among the three cold herbs, DH and HQ share similar structures and separated with Con alongside PC1. HL ran the farthest distance, with Con among the three cold herbs and was separated with Con alongside PC2. According to the most significant changes in the microbiome structure in the HL group, we can claim that the clear separation of the long-term Cold TCM group from the control group is mainly contributed by HL. As shown in Fig. 2, long-term intervention of Hot TCM had only a minor impact on the relative abundance of gut microbiota at the phylum level, except for the reduced Desulfobacterota in GJ and FZ treated mice, while HL treatment resulted in increases in Verrucomicrobiota and Campilobacterota, and depletion of Deferribacterota. At the genus level, the relative abundance of *g__Muribaculum* was increased, and *g__Dubosiella* was decreased by both Hot_LT and Cold_LT. Four genera were specifically regulated by Hot_LT including *g_parabacterodies* and *g_desulfovibrio*, and 13 genera were significantly changed by Cold_LT including *g_faecalibaculum* and *g_alloprevotella* (Fig. 2f, Additional file 2: Fig. S2A).

We next analyzed the impact on functions of the gut microbiota using PICRUSt2 analysis. In contrast to the functional changes induced by the short-term TCM intervention, there were 4 and 65 pathways associated with the long-term Hot or Cold TCM intervention respectively, but no shared pathway (Additional file 2: Fig. S2B). Most of the changed pathways belonged to metabolism and human disease (Fig. 2g, Additional file 2: Fig. S2C). The results suggested that long-term TCM intervention with Hot nature exerted relatively minor impacts on the composition and functions of the gut microbiota, but Cold TCM substantially influenced the composition of the gut microbiota, especially in terms of microbiome functions.

### The divergent compositional and functional changes of the gut microbiome after short-term or long-term TCM intervention

**Fig. 3:**
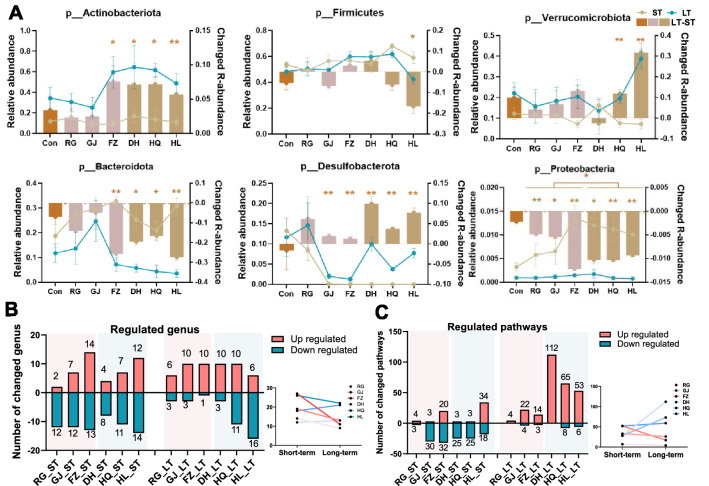
The nature-specific and time-depend regulation of TCM on gut microbiome. **A** The expression and variation of top six phylum in each group. The brown column shows the abundance in long-term experiment minus the abundance in short-term experiment. **B**, **C** The regulated genus, and pathways in each group under two timepoints. N = 5 each group, Students’ *t*-test *P < 0.05, **P < 0.01

To compare the influence on the composition of gut microbiota of short-term or long-term TCM intervention, conjoint analysis based on 16S rDNA sequencing data was adopted. The variations of the major 6 phylum under short-term or long-term TCM interventions are shown in Fig. 3a, including Actinobacteriota, Firmicutes, Verrucomicrobiota, Bacterodiota, Desulfobacterota and Proteobacteria. The long-term Cold TCM intervention resulted in a universally increased abundance of Actinobacteriota and Desulfobacterota, but reduced Bacterodiota and Proteobacteria. Interestingly, the impact of the long-term FZ intervention on Actinobacteriota, Bacterodiota and Proteobacteria was similar with Cold TCM. This may due to the high alkaloid components in FZ, which is widely associated with cold drugs. In addition, long-term HQ and HL treatment induced the same trend of changes in Firmicutes and Verrucomicrobiota. In general, the number of altered genera was reduced after long-term TCM intervention compared to the short-term group interventions in the Hot TCM and HL group, but was increased in the DH and HQ group (Fig. 3b).

In order to compare the impact on gut microbial functions, the number of altered pathways after short- or long-term TCM interventions were analyzed based on 16S rDNA gene sequencing data (Fig. 3c). The altered pathways were 7, 33 and 52 in the short-term RG, GJ and FZ groups, and 28, 28 and 52 in the DH, HQ and HL groups, respectively. The majority of altered pathways were downregulated by the short-term TCM intervention, except for FZ and HL. In contrast, long-term Hot TCM treatment resulted in 4, 26 and 17 altered pathways by RG, GJ and FZ, and most of them were upregulated. However, long-term Cold TCM treatment induced a dramatic increase in the number of altered pathways, and most of them were upregulated. In summary, the impact of TCM interventions on microbial functions were TCM nature- and time-related. The influence on gut microbial functions were enhanced by long-term treatment with Cold TCM than with a short-term intervention. Although the number of altered pathways was decreased in long-term Hot TCM treated mice compared to their short-term partners, the alteration trend was opposite in Cold TCM between the two time points.

### Short-term TCM intervention altered the serum metabolic profile

**Fig. 4:**
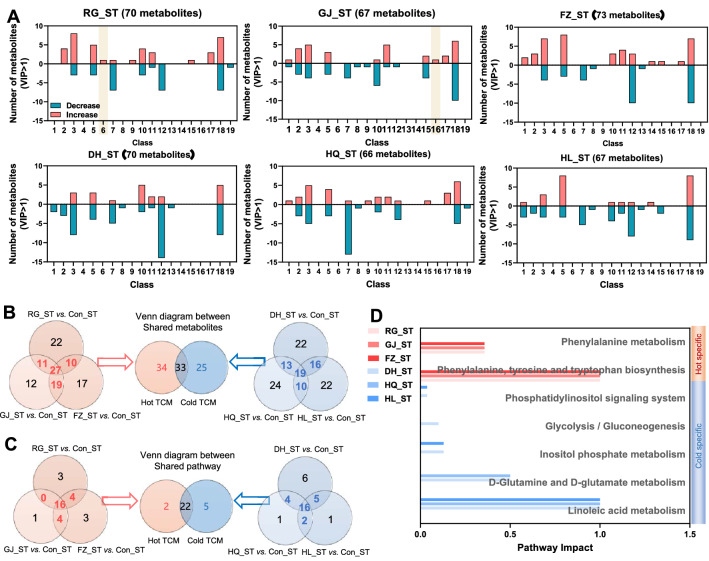
The impact of TCM short-term intervention on serum metabonomic. **A** The classification of metabolites with VIP > 1 in each group. **B** Venn diagram between Hot_ST and Cold_ST based on shared metabolites of each nature. **C** Venn diagram between Hot_ST and Cold_ST based on shared pathways by metabolic pathway enrichment analysis on KEGG pathway level 3 with pathway impact > 0. **D** The pathway impact of specific pathways in Hot_ST and Cold_ST. Class 1 is alcohols, 2 is alkylamines, 3 is amino acids, 4 is benzoic acids, 5 is carbohydrates, 6 is carnitines, 7 is fatty acids, 8 is indoles, 9 is inorganic oxide, 10 is lipids, 11 is nucleotides, 12 is organic acids, 13 is organooxygen compound, 14 is peptides, 15 is phenols, 16 is phenyl propanoic acids, 17 is pyridines, 18 is reaction ratio, and 19 is vitamins

Given the link between the gut microbiota and host metabolism [33], we further compared the impact of short-term TCM intervention with either Hot or Cold nature on the serum metabolic profile using untargeted metabolomics. With the criteria of variable importance in projection (VIP) > 1, the short-term intervention of RG, GJ and FZ induced changes of 70, 67, and 73 in serum metabolites, while DH, HQ and HL induced 70, 66 and 67 metabolites change respectively (Fig. 4a, Additional file 4: Fig. S4A). The short-term intervention of RG regulated carnitine metabolites specifically, and the phenyl propanoic acids class in the GJ group. Venn analysis was performed to explore further the differences between Hot and Cold TCM (Fig. 4b). Hot TCM induced 67 changed metabolites, while 58 metabolites were regulated by Cold TCM, most of them being amino acids, organic acid and carbohydrates. The fourth metabolite category was lipids in Hot TCM with a proportion of 10.45%, and fatty acids in Cold TCM with a proportion of 12.07% (Additional file 4: Fig. S4B). Thirty-three metabolites were commonly altered in both groups.

Pathway enrichment analysis was employed to explore changes in biological functions. Hot TCM regulated 2 metabolic pathways specifically while 5 pathways were regulated by Cold TCM according to the Venn analysis; most pathways were regulated by both of them (Fig. 4c, d, Additional file 4: Fig. S4C, D). It was noticed that Hot_ST had a more extensive disturbance effect on synthesis and decomposition of amino acids, while Cold_ST regulated glucose and lipid metabolism, like glycolysis/gluconeogenesis, linoleic acid metabolism and d-glutamate metabolism. These results suggested that a short-term TCM intervention with two properties exerted relatively major impacts on serum metabolites, but minor influences on the function of metabolites, especially for Hot_ST.

### Serum metabolic profile after a long-term TCM intervention

**Fig. 5:**
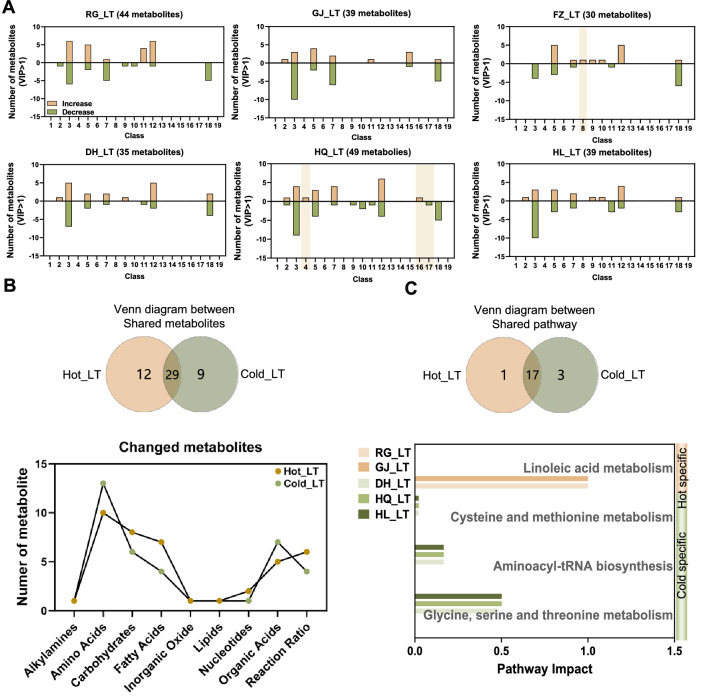
The impact of TCM long-term intervention on serum metabonomic. **A** The classification of metabolites with VIP > 1 in each group. **B** Venn diagram between Hot_LT and Cold_LT based on shared metabolites of each nature, and the number of changed metabolites in each class. **C** Venn diagram based on Hot_LT shared pathway and Cold_LT shared pathway by metabolic pathway enrichment analysis on KEGG pathway level 3 with pathway impact > 0, and bar chart shows the pathway impact of specific pathways. Metabolite classes as in Fig. 4

Next, to explore the long-term impact of the TCM intervention on host metabolism, mice received 35 days TCM treatment with a Hot or Cold nature. After the long-term intervention, the number of screen metabolites was reduced in 6 groups meeting the condition of VIP > 1 compared to their short-term partners (Figs. 4a, 5a). The serum concentration of indole metabolites was increased by FZ_LT, while serum concentrations of benzoic acid and phenyl propanoic acid metabolites were increased, while pyridines metabolites were decreased by HQ_LT. Based on the Venn analysis, most of the changed metabolites in Hot_LT or Cold_LT were included in amino acids, but the second metabolite class was fatty acids (proportion of metabolites, 17.07%) in Hot_LT and organic acid (18.42%) in Cold_LT (Fig. 5b, Additional file 5: Fig. S5B).

Corresponding to pathway enrichment analysis of the short-term intervention, most pathways were regulated by both Hot_LT and Cold_LT (Fig. 5c, Additional file 5: Fig. S5C, D). Also, the linoleic acid metabolism pathway was specific reduced by Hot_LT, and Cold_LT specifically regulated the glycine, serine and threonine metabolism pathways, the aminoacyl-tRNA biosynthesis pathway, and the cysteine and methionine metabolism pathways. Together, serum metabonomic variety by TCM was property specific and narrowed with a prolonged intervention.

### The regulation of serum metabolomic by TCM is nature-specific and time-related

**Fig. 6:**
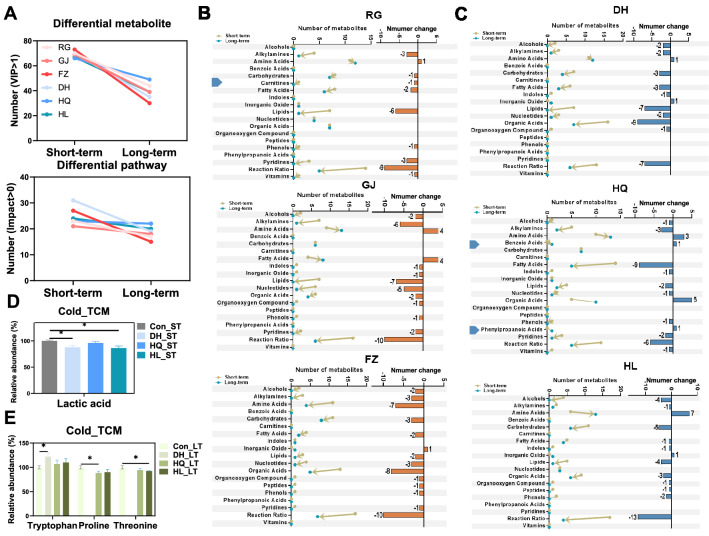
The different changes of serum metabolites over time in Hot- and Cold-TCM treated group. **A** The number of regulated metabolites with condition of VIP > 1 and pathways with condition of impact > 0 in each group under two timepoints. **B**, **C** The metabolites number of each class in each group under two timepoints. Triangles on the left indicates specific changed class in Hot_ or Cold_TCM. **D**, **E** The significantly changed metabolites which involved in specific changed pathways in Cold_ST and Cold_LT. N = 5 each group, Student’s *t*-test *P < 0.05, **P < 0.01

To evaluate further the impact of TCM on host metabolism, time-cross analysis based on serum metabolomic data was employed. The number of changed metabolites and pathways were found to be reduced along with extension of the intervention time in two properties (Fig. 6a–e). Hot TCM and Cold TCM had different impacts on metabolites class with an increase in the intervention time (Fig. 6b, c). HQ specifically increased the metabolite numbers of the benzoic acid and phenyl propanoic acid classes, and RG specific decreases in the metabolite numbers of the carnitines class. The metabolite numbers of amino acids, fatty acids and inorganic oxide classes were found to be regulated inversely by two properties. Based on KEGG pathway enrichment and Venn analysis, Hot_ST, Cold_ST, Hot_LT and Cold_LT regulated 2, 5, 1 and 3 pathways respectively, and the metabolites included in each pathway are shown in Additional file 6: Fig. S6, which were suspected to play a constructive role in distinguishing Hot TCM from Cold TCM. In the short-term intervention (Additional file 6: Fig. S6A), the 3 Hot TCM groups had reduced the concentration of l-tyrosine and increased the concentration of l-phenylalanine, which included the phenylalanine, tyrosine and tryptophan biosynthesis pathways, and the phenylalanine metabolism pathway. DH and HL significantly reduced the relative abundance of lactic acid involved in the glycolysis/gluconeogenesis pathway (Fig. 6d). In the long-term intervention (Additional file 6: Fig. S6B, Fig. 6e), linoleic acid was associated with the linoleic acid metabolism pathway of Hot_LT and 12 metabolites were including in 3 pathways of Cold_LT. Tryptophan concentrations were significantly increased by DH, proline was decreased by HQ, and threonine was decreased by HL. In addition, the presence or absence of carnitine, benzoic acid and phenyl propanoic acid classes may be used to classify Hot and Cold TCM.

### Correlation analysis between altered gut microbiota and serum metabolites

**Fig. 7:**
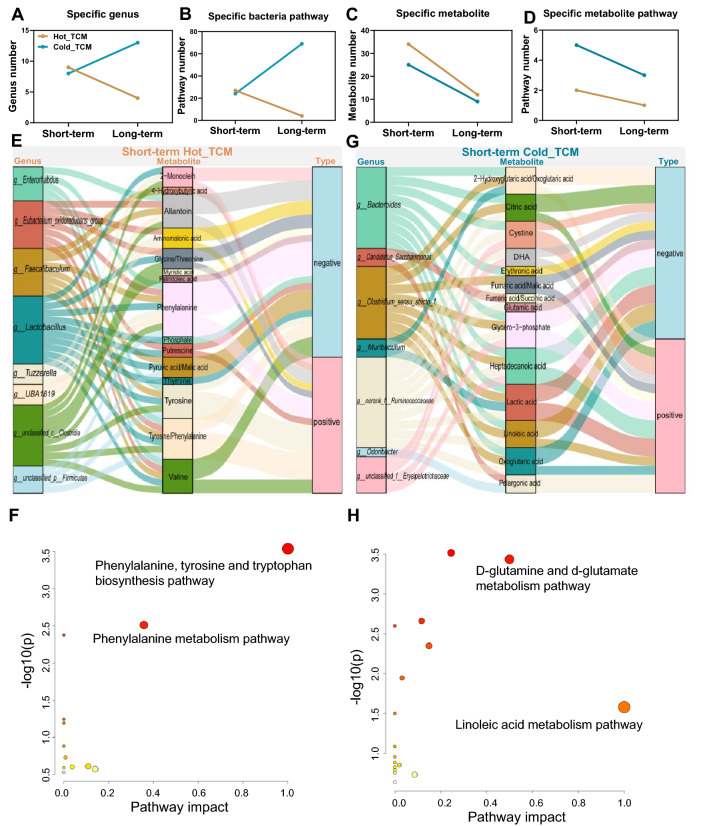
The relevance analysis between specific changed genus and specific changed metabolites in each property at short-term intervention. **A–D** The number of specific genera, specific bacteria pathways, specific metabolites, and specific metabolic pathways in Hot_ and Cold_TCM under two timepoints. **E**, **G** The relevance analysis between specific changed genera and specific changed metabolites in each property at short-term intervention under spearman analysis. Only significantly associations are presented in the figure. **F**, **H** KEGG pathway enrichment analysis based on metabolites in **E** and **G** which are significantly correlated with genus. N = 5 each group

Summarizing the above changes, the number of specific genera, bacteria pathways, metabolites and metabolite pathways was decreased in Hot_LT compared with Hot_ST (Fig. 7a–d). These numbers showed a different tendency in Cold_LT when compared to Cold_ST. In the short-term intervention, specific genus and bacteria pathway numbers were similar during Cold TCM compared to Hot TCM, but these numbers were surprisingly increased during Cold_LT (Fig. 7a, b). Despite Cold TCM maintaining the same change tendency of specific metabolite and metabolites pathway numbers with Hot TCM, the numbers in Cold TCM were much bigger than for Hot TCM at two time points (Fig. 7c, d), suggesting a wider body metabolism effect after the Cold TCM intervention.

Given the strong association of serum metabolites with the microbiome, a correlation analysis based on a changed gut microbiota (specific differential genus with *P* < 0.05) and changed serum metabolites (specific differential metabolites with VIP > 1) was carried out with the condition of the correlation *P* < 0.05. After the short-term intervention, 8 genera were significantly associated with 15 serum metabolites in Hot TCM (Fig. 7e). Pathway enrichment analysis of these 15 metabolites revealed two pathways which were the same as Hot_ST specific pathways that are represented in Fig. 4e, and the metabolites phenylalanine and tyrosine were associated with these two pathways (Fig. 7f). The correlation of phenylalanine with *g__Enterorhabdus*, *g__Eubacterium_oxidoreducens_group*, *g__Faecalibaculum*, *g__Lactobacillus* and *g__Tuzzerella* was negative, with *g__UBA1819*, *g__unclassified_c__Clostridia*, *g__unclassified_p__Firmicutes* was positive, while tyrosine was negatively associated with *g__unclassified_p__Firmicutes*, but positively associated with *g__Tuzzerella*, *g__unclassified_c__Clostridia*, *g__Lactobacillus*, and *g__UBA1819*. These results suggested that the changes in the gut microbiome may partly induce the specific alterations in serum metabolites and functions. Our conjecture was verified in subsequent correlation analyses.

In Cold TCM, there were 7 genera associated with 14 metabolites, and these metabolites were evaluated in pathway enrichment analysis. Two of 8 pathways obtained by enrichment (impact > 0) were coincident with the Cold_ST specific pathway (Figs. 4e, 7g, h). Glutamic acid and oxo glutaric acid were included in the d-glutamine and d-glutamate metabolism pathway. Oxo glutaric acid was positively correlated with *g_norank_f_Ruminoccoccaceae,* which was negatively correlated with glutamic acid. Also, oxo glutaric acid was negatively correlated with *g__Bacterodiess* and *g__Clostridium_sensu_stricto_1*. Linoleic acid which was clearly involved in the linoleic acid metabolism pathway was negatively associated with *g__Bacteroides* and *g__Clostridium_sensu_stricto_1*, and positively associated with *g_norank_f_Ruminoccoccaceae*.

**Fig. 8:**
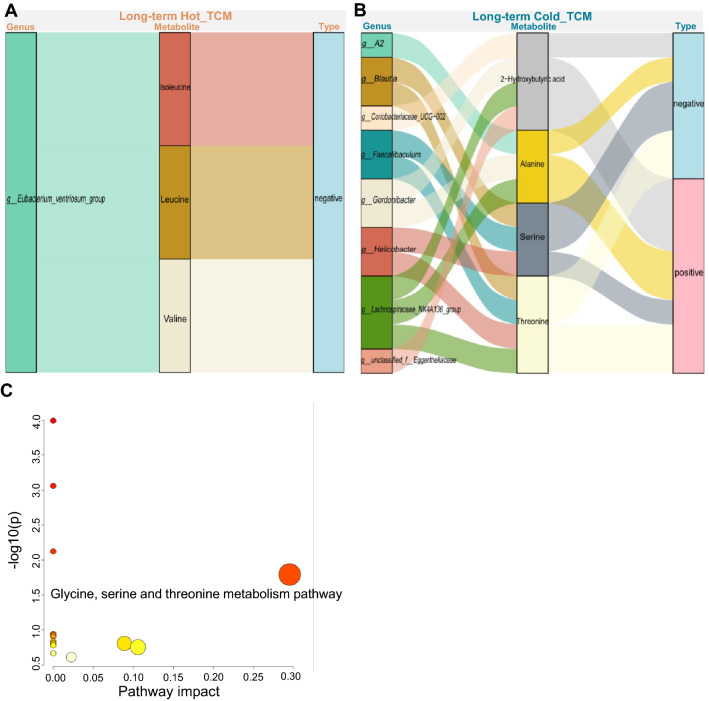
The relevance analysis between specific changed genus and specific changed metabolites in each property at long-term intervention. **A**, **B** The relevance analysis between specific changed genera and specific changed metabolites in each property at long-term intervention under spearman analysis. Only significantly association would be presented in the figure. **C** KEGG pathway enrichment analysis based on metabolites in (**B**) which are significantly correlated with genus. N = 5 each group

In Hot_LT, only the *g__Eubacterium_ventriosum_group* was negatively associated with isoleucine, leucine and valine. This finding may be attributed to the significant reduction in genus numbers (Fig. 8a), shown by the slight effect of Hot_LT on host metabolism. In Cold_LT, 8 genera were significantly associated with 4 metabolites (Fig. 8b), and KEGG pathway enrichment indicated that Cold_LT specifically regulated the pathways involved in glycine, serine and threonine metabolism (impact > 0, Fig. 8c), which also involved the metabolite threonine. Threonine was negatively associated with *g__Gordonibacter* and *g__A2,* and positively associated with *g__Monoglobus* and *g__norank_f__Eggerthellaceae*. These results partly verified our conjecture, and also revealed different changes in the microbiome and in metabolism caused by Hot TCM and Cold TCM.

## Discussion

Thousands of years have passed since Cold and Hot properties were regarded as one of the guiding principles of TCM when used to treat disease. TCM classified herbs into different natures like Cold and Hot to treat diseases with opposite natures, and knowledge developed with continuing experience in clinical practice. However, using modern language, especially developed muti omics, to reveal the specific host differences induced by different nature herbs was the necessary approach to improve the scientific interpretation and wide application of TCM. Therefore, we investigated the divergent impacts of TCM with either Cold or Hot properties on the gut microbiome and host metabolism in the context of a short- (7 days) or long-term (35 days) intervention in mice. The results revealed that either a Hot or Cold TCM intervention exerted obvious influences on the gut microbiome and the serum metabolic profile, and those changes in the microbial activity or metabolism was narrowed with time.

The theory of medicinal properties is one of the core theories of TCM, which is an important basis for guiding the clinical application of TCM [34]. It is usually recognized that TCMs with different properties divergently influence the functions of the central nervous system, sympathetic nerves and the endocrine system, basal metabolic rate and secretion of cytokines [35]. The Hot TCM, like RG, GJ and FZ has the capacity to increase body temperature [36, 37], which is a property usually used to cure nervous system diseases [38], and depression [39]. Cold TCM such as DH, HQ and HL has the opposite effect on body temperature [40], and is used to improve depressive-like behavior [41], and has the capacity to regulate energy metabolism and improve mitochondrial functions [42]. Given the holistic effect of TCM on the host, a single parameter cannot reflect its systemic impact on host metabolism. Therefore, omics approaches, especially metabolomics have been increasingly applied in research on TCM actions, and the general properties of TCMs. Meanwhile, the modulating effects of TCM on the gut microbiome has attracted more attention due to the fact that complex components within orally administered TCM extracts usually have profound impacts on the gut microbiome [43]. Although there have been studies using modern technology to explore the effects of Cold or Hot TCM drugs on animal liver energy metabolism or body temperature, there is still a paucity of articles comparing the effects of Cold and Hot drugs on host metabolism. Therefore, in the current study, we adopted combined gut microbiome and serum metabolomics to investigate the impacts of TCM with either Cold or Hot properties on mice in the context of short- or long-term interventions.

In the short-term intervention, Hot or Cold TCM had different impacts on the gut microbiome structure and functions, despite the comparable number of changed genera or pathways between the two classes. Previous publications suggested that the components within Hot or Cold TCMs regulated lipid metabolism [44–46] and cAMP signaling [47–49] respectively, findings consistent with our results, including that the adipocytokine signaling pathway was activated by Hot TCM while the cAMP signaling pathway was stimulated by Cold TCM according to the PICRUSt2 analysis. Similarly, Zhang et al. studied the impact of TCM with Hot, Warm, Cool or Cold natures on the gut microbiota of C57BL/6J mice with 4 weeks interventions. They found dramatic changes in the gut microbiota with Cold TCM but not with Hot TCM [19], suggesting that Cold TCM had a greater impact on the gut microbiome compared to Hot TCM, findings consistent with the present results. Although extremely complicated and poorly understood, the diversified actions of TCM undoubtedly involve complex chemical components and their interactions. Similarly, the nature of Hot or Cold TCM is associated with their chemical components. In Cold TCM, the main active element of DH is gallic acid, which can be absorbed in the intestinal tract producing extensive effects on host metabolism [50]. However, baicalin and berberine, the main ingredients of HQ and HL, have many biological functions but it is noteworthy that they have only low absorption [51, 52]. Much evidence suggests that a role of the gut microbiota as targets for the multifunctional role of baicalin and berberine [53, 54]. These data echo our current findings, that is, the influence of Cold TCM on the gut microbiome was more obvious than Hot TCM, and was increased by the long-term intervention, but opposite to Hot TCM effects. In addition to the analysis of the gut microbiome and individual serum metabolite profiles, correlation analyses between altered gut microbiota and serum metabolites were performed. In Hot_ST correlation analysis, it was found that decreased *g__Faecalibaculum* and *g__Lactobacillus* were negatively associated with the ratio of pyruvic acid to malic acid. Interestingly, in Cold_ST, the increased genus *g__Candidatus_Saccharimonas* and *g__norank_f__Ruminococcaceae* were positively related to fumaric acid/succinic acid and fumaric acid/malic acid, respectively. The decreased genus *g__Clostridium_sensu_stricto_1* was negatived related to fumaric acid/malic acid reflecting the disorder of the TCA cycle metabolic pathway. The results indicated that the differential regulation of Cold and Hot TCM on body temperature published in previously publications may be effected by intestinal bacteria changing the TCA cycle [7, 35].

Also, the pathway enrichment analysis based on metabolites which were significantly connected with the genus revealed that changes in the phenylalanine, tyrosine and tryptophan biosynthesis pathways and the phenylalanine metabolism pathway in Hot_ST, and changes of d-glutamine, d-glutamate and linoleic acid metabolism pathways in Cold_ST, and changes in glycine, serine and threonine metabolism pathways in Cold_LT were all strongly associated with the regulation of TCM on the gut microbiota. Therefore, we speculated that the differential regulation of Hot or Cold TCM on serum metabolic pathways was associated with the modulation of gut microbiota. These results indicated that an association of gut bacteria with serum metabolism provides a new perspective to understand the different metabolism impacts of Hot and Cold TCM.

Although, we took the opportunity to investigate the time dependent effects of TCMs with different properties using multi omics, there were still some limitations to our research. First, since there are hundreds of phytomedicines commonly used in the clinic and therefore results based on six TCM could not represent all TCM options for therapy. Thus, more TCM research is necessary. Second, our research was based only on normal C57BL/6J mice. More animal species, such as the rat or an illness model should be used in future research. Finally, serum metabonomic was our evaluation criterion for host metabolism as it brings together all the metabolites, but more indicators, such as changes in biochemical and liver indexes should be considered in future studies.

## Conclusions

The current study indicated that TCM with Hot or Cold properties exerted profound effects on the host metabolism or gut microbiome in composition and function. These influences were changed with the intervention period, in which long-term intervention with Cold TCM resulted in a greater impact than Hot TCM, suggesting a different adaptation of host metabolism or the gut microbiome to a TCM intervention. The results highlighted the potential of characterizing TCM natures with the gut microbiome and metabolomics. However, further investigations are warranted to reveal the common or unique characteristics that are intricately associated with the Hot and Cold properties of TCM, by adopting more medicines and their application in disease models or TCM formulae.

## Supplementary Information

**Additional file 1. Fig. S1**: **A** Heatmap shown the specific genus and shared genus between two natures. **B** Heatmap shown 11 Hot_ST specific pathways, eight Cold_ST specific pathways, and 16 shared pathways at KEGG pathway level 3.

**Additional file 2. Fig. S2**: **A** Venn diagram between Hot_LT and Cold_LT based on shared genus in each nature. **B** Venn diagram shows the overlap between Hot TCM shared pathway and Cold TCM shared pathway by PICRUSt2 analysis on KEGG pathway level 3 under Mann–Whitney U test. **C** Heatmap shown the four Hot TCM specific pathways and 65 Cold TCM specific pathways at KEGG level 3 by PICRUSt2.

**Additional file 3. Fig. S3**: **A** Volcano map shows the distribution of metabolites in each group. Red spots presented down-regulated metabolites, while blue spots mean up-regulated metabolites under the condition VIP > 1. **B** The number of changed metabolites in each class in Hot_ST and Cold_ST. **C** Under enrichment analysis, the pathways satisfying the conditional impact > 0 in each group.

**Additional file 4. Fig. S4**: **A** Volcano map shows the distribution of metabolites in each group. Red spots presented down-regulated metabolites, while blue spots mean up-regulated metabolites under the condition VIP > 1. **B** Venn diagram between Hot_LT and Cold_LT based on shared metabolites of each nature. **C** Venn diagram between Hot_LT and Cold_LT based on shared pathways by metabolic pathway enrichment analysis on KEGG pathway level 3 with condition of pathway impact > 0. **D** Under enrichment analysis, the pathways satisfying the conditional impact > 0 in each group.

**Additional file 5. Fig. S5**: **A**, **B** The metabolites involved in specific changed pathways in Hot_ and Cold_TCM. N = 5 each group, Student’s *t*-test *P < 0.05, **P < 0.01.

**Additional file 6. Fig. S6**: The main peaks of six extracts. *Copitdis Rhizoma* was determined by UV chromatograph due to the instable baseline under TOF-MS/MS, others were determined by mass chromatograph. The names and proportions of peaks are shown in Table 1.

## Data Availability

Sequence data associated with this project have been deposited in the NCBI Short Read Archive (PRJNA832599, PRJNA832616) database.

## Abbreviations

16S rDNA: 16S ribosomal deoxyribonucleic acid
DH: Dahuang for Chinese, *Rhei Radix et Rhizoma* for Latin name
DNA: Deoxyribonucleic acid
FZ: Fuzi for Chinese, *Aconiti Lateralis Radix Praeparata* for Latin name
GC–MS: Gas chromatography–tandem mass spectrometry
GC–TOF/MS: Fast gas chromatography–time of flight mass spectrometry
GJ: Ganjiang for Chinese, *Zingiberis Rhizoma* for Latin name
HL: Huanglian for Chinese, *Copitdis Rhizoma* for Latin name
HQ: Huangqin for Chinese, *Scutellariae Radix* for Latin name
KEGG: Kyoto Encyclopedia of Genes and Genomes
OTU: Operational taxonomic units
RG: Rougui for Chinese, *Cinnamomi Cortex*l for Latin name
RNA: Ribonucleic acid
TCM: Traditional Chinese Medicine
UPLC–QTOF-MS/MS: Ultrahigh performance liquid chromatography coupled with quadrupole time flight mass spectrometry
VIP: Variable importance in projection
