# Supplementary material for: Divergent impacts on the gut microbiome and host metabolism induced by traditional Chinese Medicine with Cold or Hot properties in mice
Source: Chin Med. 2022 Dec 26;17:144. doi: 10.1186/s13020-022-00697-2 (PMC9793677; doi:10.1186/s13020-022-00697-2)
Supplement: Supplementary file 1 — Additional file 1. Fig. S1: A Heatmap shown the specific genus and shared genus between two natures. B Heatmap shown 11 Hot_ST specific pathways, eight Cold_ST specific pathways, and 16 shared pathways at KEGG pathway level 3. [file 13020_2022_697_MOESM1_ESM.pptx]

## Slide 1
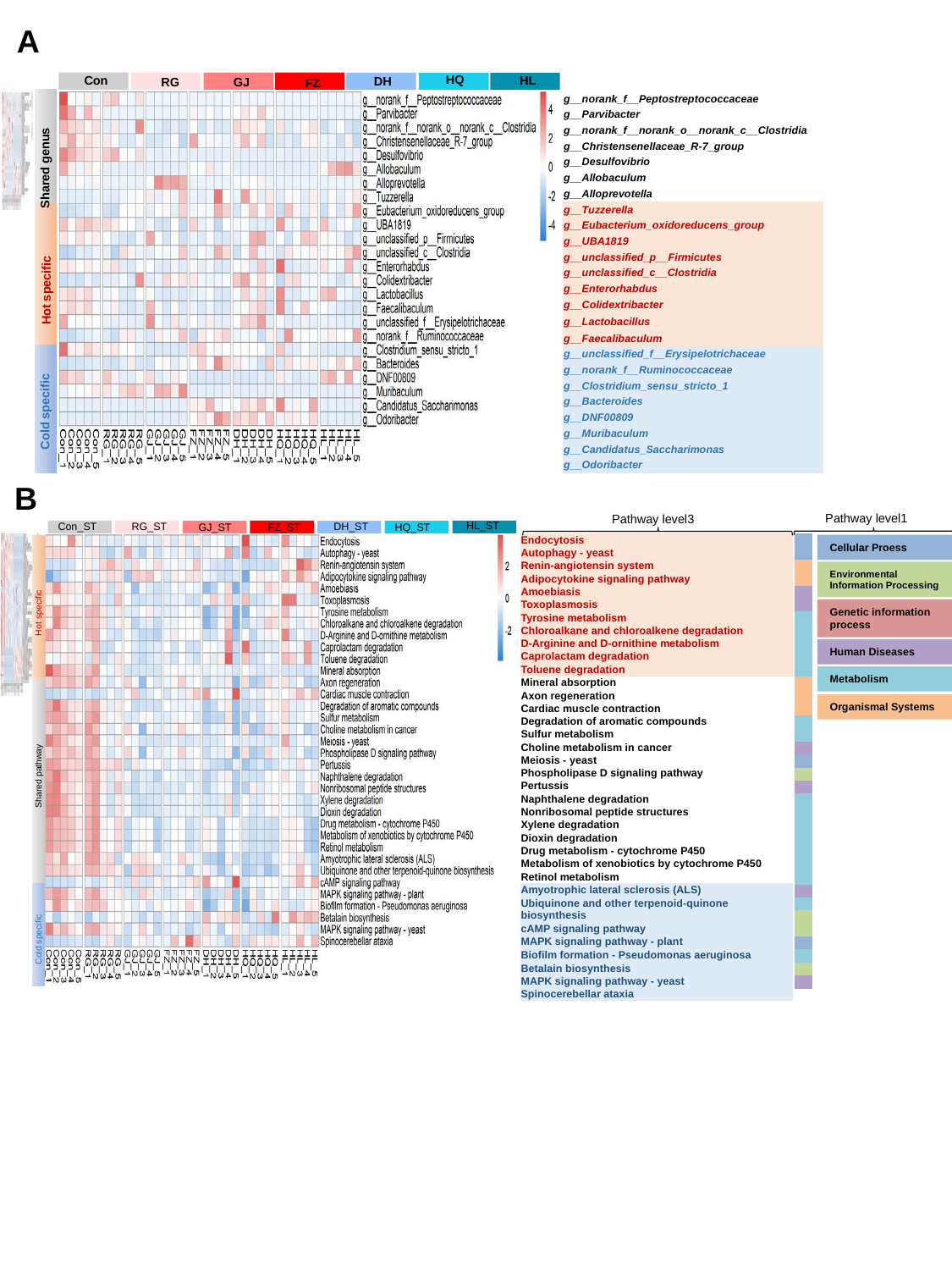

A
HQ
Con
HL
DH
RG
GJ
FZ
| g\_\_norank\_f\_\_Peptostreptococcaceae |
| --- |
| g\_\_Parvibacter |
| g\_\_norank\_f\_\_norank\_o\_\_norank\_c\_\_Clostridia |
| g\_\_Christensenellaceae\_R-7\_group |
| g\_\_Desulfovibrio |
| g\_\_Allobaculum |
| g\_\_Alloprevotella |
| g\_\_Tuzzerella |
| g\_\_Eubacterium\_oxidoreducens\_group |
| g\_\_UBA1819 |
| g\_\_unclassified\_p\_\_Firmicutes |
| g\_\_unclassified\_c\_\_Clostridia |
| g\_\_Enterorhabdus |
| g\_\_Colidextribacter |
| g\_\_Lactobacillus |
| g\_\_Faecalibaculum |
| g\_\_unclassified\_f\_\_Erysipelotrichaceae |
| g\_\_norank\_f\_\_Ruminococcaceae |
| g\_\_Clostridium\_sensu\_stricto\_1 |
| g\_\_Bacteroides |
| g\_\_DNF00809 |
| g\_\_Muribaculum |
| g\_\_Candidatus\_Saccharimonas |
| g\_\_Odoribacter |
Shared genus
Hot specific
Cold specific
B
Pathway level1
Pathway level3
HL_ST
Con_ST
RG_ST
DH_ST
GJ_ST
HQ_ST
FZ_ST
Cellular Proess
Hot specific
Shared pathway
Cold specific
Environmental Information Processing
Genetic information process
Human Diseases
Metabolism
Organismal Systems
| Endocytosis |
| --- |
| Autophagy - yeast |
| Renin-angiotensin system |
| Adipocytokine signaling pathway |
| Amoebiasis |
| Toxoplasmosis |
| Tyrosine metabolism |
| Chloroalkane and chloroalkene degradation |
| D-Arginine and D-ornithine metabolism |
| Caprolactam degradation |
| Toluene degradation |
| Mineral absorption |
| Axon regeneration |
| Cardiac muscle contraction |
| Degradation of aromatic compounds |
| Sulfur metabolism |
| Choline metabolism in cancer |
| Meiosis - yeast |
| Phospholipase D signaling pathway |
| Pertussis |
| Naphthalene degradation |
| Nonribosomal peptide structures |
| Xylene degradation |
| Dioxin degradation |
| Drug metabolism - cytochrome P450 |
| Metabolism of xenobiotics by cytochrome P450 |
| Retinol metabolism |
| Amyotrophic lateral sclerosis (ALS) |
| Ubiquinone and other terpenoid-quinone biosynthesis |
| cAMP signaling pathway |
| MAPK signaling pathway - plant |
| Biofilm formation - Pseudomonas aeruginosa |
| Betalain biosynthesis |
| MAPK signaling pathway - yeast |
| Spinocerebellar ataxia |
