# Supplementary material for: Divergent impacts on the gut microbiome and host metabolism induced by traditional Chinese Medicine with Cold or Hot properties in mice
Source: Chin Med. 2022 Dec 26;17:144. doi: 10.1186/s13020-022-00697-2 (PMC9793677; doi:10.1186/s13020-022-00697-2)
Supplement: Supplementary file 2 — Additional file 2. Fig. S2: A Venn diagram between Hot_LT and Cold_LT based on shared genus in each nature. B Venn diagram shows the overlap between Hot TCM shared pathway and Cold TCM shared pathway by PICRUSt2 analysis on KEGG pathway level 3 under Mann–Whitney U test. C Heatmap shown the four Hot TCM specific pathways and 65 Cold TCM specific pathways at KEGG level 3 by PICRUSt2. [file 13020_2022_697_MOESM2_ESM.pptx]

## Slide 1
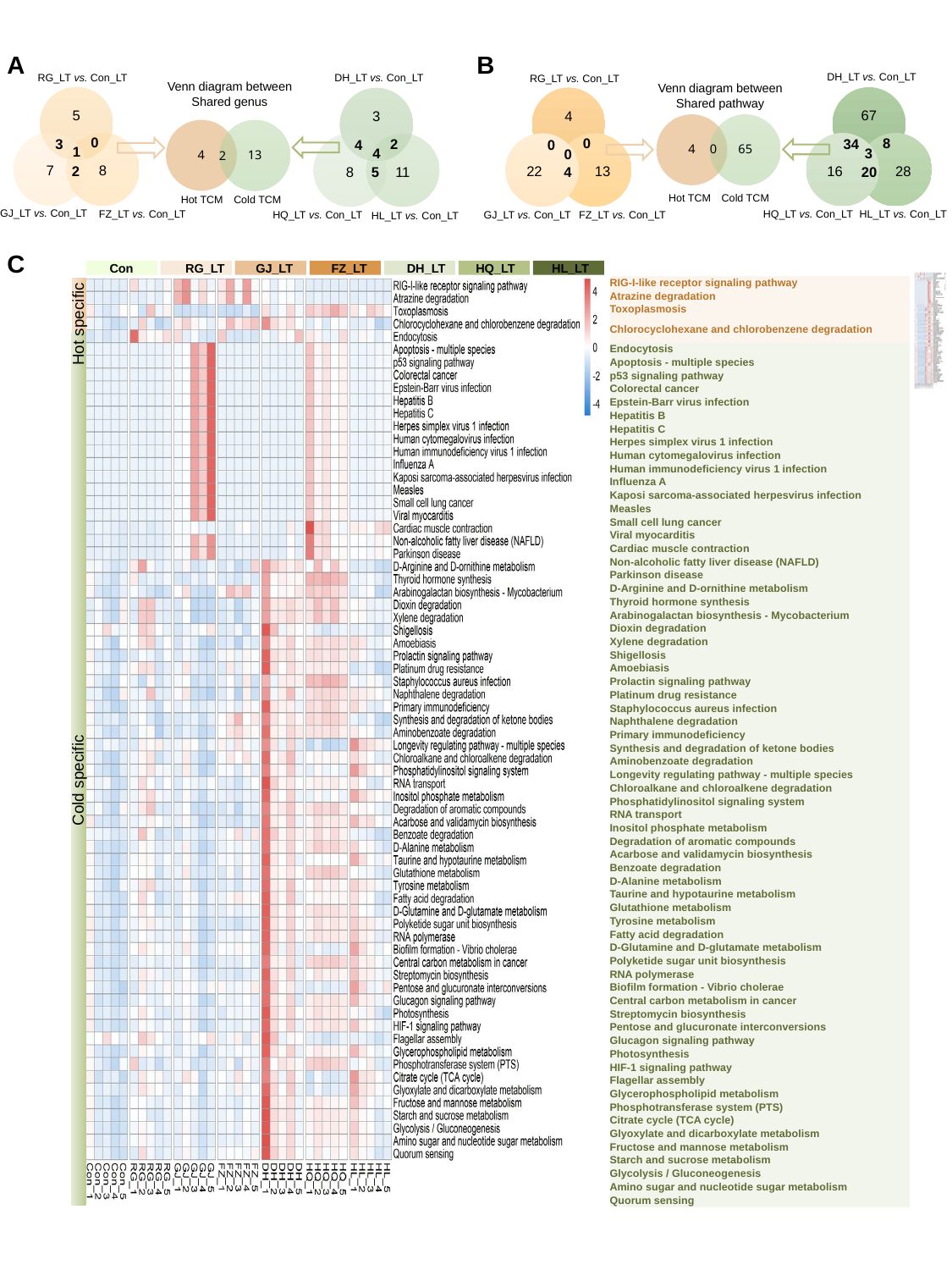

B
A
DH_LT vs. Con_LT
DH_LT vs. Con_LT
RG_LT vs. Con_LT
RG_LT vs. Con_LT
Venn diagram between Shared genus
Venn diagram between Shared pathway
67
16
28
4
22
13
0
8
0
2
3
34
0
4
0
1
3
4
0
2
2
20
4
5
Cold TCM
Hot TCM
Cold TCM
Hot TCM
GJ_LT vs. Con_LT
FZ_LT vs. Con_LT
HQ_LT vs. Con_LT
HL_LT vs. Con_LT
FZ_LT vs. Con_LT
HQ_LT vs. Con_LT
GJ_LT vs. Con_LT
HL_LT vs. Con_LT
C
Con
RG_LT
GJ_LT
FZ_LT
DH_LT
HQ_LT
HL_LT
| RIG-I-like receptor signaling pathway |
| --- |
| Atrazine degradation |
| Toxoplasmosis |
| Chlorocyclohexane and chlorobenzene degradation |
| Endocytosis |
| Apoptosis - multiple species |
| p53 signaling pathway |
| Colorectal cancer |
| Epstein-Barr virus infection |
| Hepatitis B |
| Hepatitis C |
| Herpes simplex virus 1 infection |
| Human cytomegalovirus infection |
| Human immunodeficiency virus 1 infection |
| Influenza A |
| Kaposi sarcoma-associated herpesvirus infection |
| Measles |
| Small cell lung cancer |
| Viral myocarditis |
| Cardiac muscle contraction |
| Non-alcoholic fatty liver disease (NAFLD) |
| Parkinson disease |
| D-Arginine and D-ornithine metabolism |
| Thyroid hormone synthesis |
| Arabinogalactan biosynthesis - Mycobacterium |
| Dioxin degradation |
| Xylene degradation |
| Shigellosis |
| Amoebiasis |
| Prolactin signaling pathway |
| Platinum drug resistance |
| Staphylococcus aureus infection |
| Naphthalene degradation |
| Primary immunodeficiency |
| Synthesis and degradation of ketone bodies |
| Aminobenzoate degradation |
| Longevity regulating pathway - multiple species |
| Chloroalkane and chloroalkene degradation |
| Phosphatidylinositol signaling system |
| RNA transport |
| Inositol phosphate metabolism |
| Degradation of aromatic compounds |
| Acarbose and validamycin biosynthesis |
| Benzoate degradation |
| D-Alanine metabolism |
| Taurine and hypotaurine metabolism |
| Glutathione metabolism |
| Tyrosine metabolism |
| Fatty acid degradation |
| D-Glutamine and D-glutamate metabolism |
| Polyketide sugar unit biosynthesis |
| RNA polymerase |
| Biofilm formation - Vibrio cholerae |
| Central carbon metabolism in cancer |
| Streptomycin biosynthesis |
| Pentose and glucuronate interconversions |
| Glucagon signaling pathway |
| Photosynthesis |
| HIF-1 signaling pathway |
| Flagellar assembly |
| Glycerophospholipid metabolism |
| Phosphotransferase system (PTS) |
| Citrate cycle (TCA cycle) |
| Glyoxylate and dicarboxylate metabolism |
| Fructose and mannose metabolism |
| Starch and sucrose metabolism |
| Glycolysis / Gluconeogenesis |
| Amino sugar and nucleotide sugar metabolism |
| Quorum sensing |
Hot specific
Cold specific
