# Supplementary material for: Divergent impacts on the gut microbiome and host metabolism induced by traditional Chinese Medicine with Cold or Hot properties in mice
Source: Chin Med. 2022 Dec 26;17:144. doi: 10.1186/s13020-022-00697-2 (PMC9793677; doi:10.1186/s13020-022-00697-2)
Supplement: Supplementary file 3 — Additional file 3. Fig. S3: A Volcano map shows the distribution of metabolites in each group. Red spots presented down-regulated metabolites, while blue spots mean up-regulated metabolites under the condition VIP > 1. B The number of changed metabolites in each class in Hot_ST and Cold_ST. C Under enrichment analysis, the pathways satisfying the conditional impact > 0 in each group. [file 13020_2022_697_MOESM3_ESM.pptx]

## Slide 1
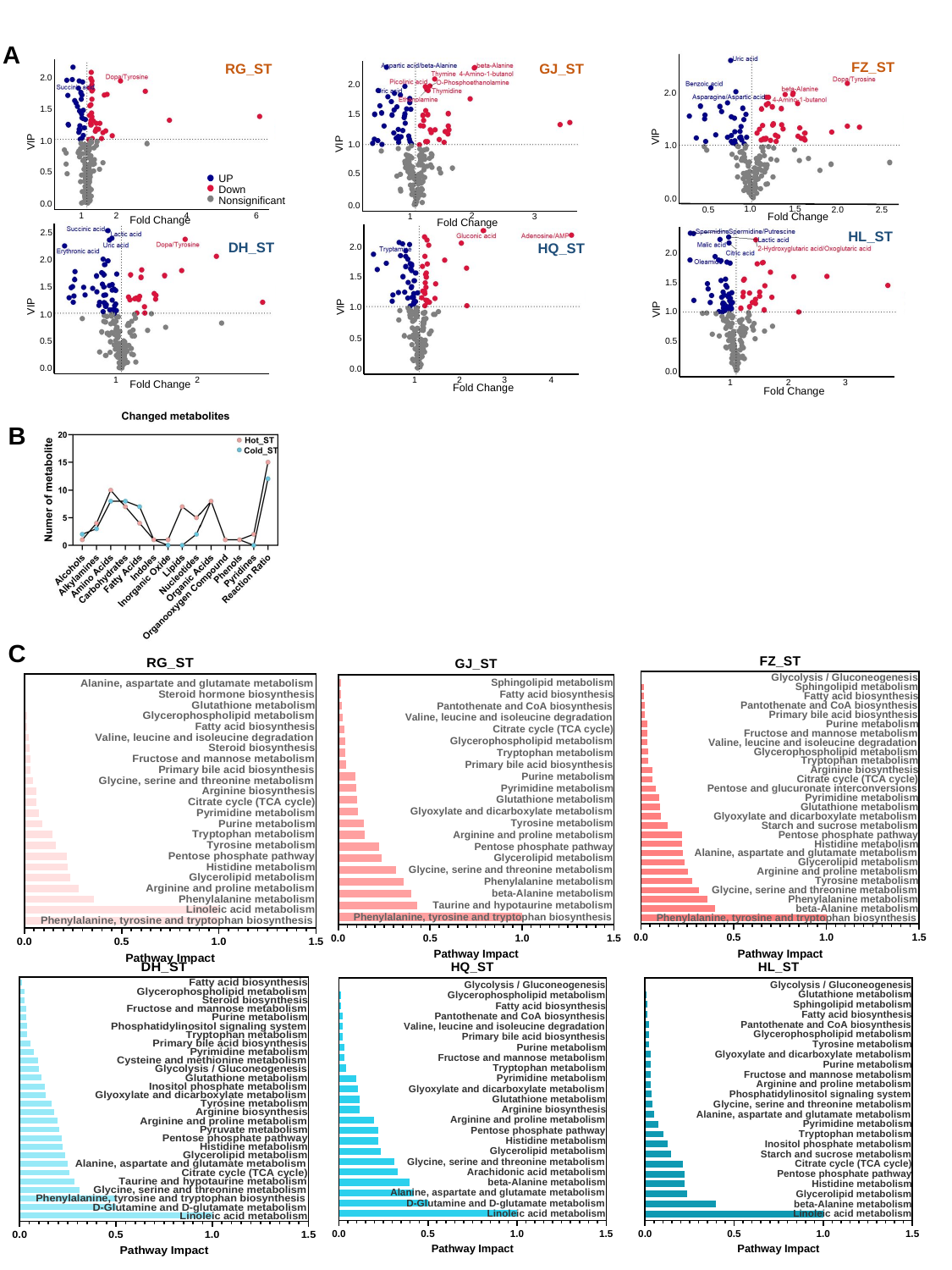

A
FZ_ST
2.0
VIP
1.0
0.0
1.0
1.5
0.5
2.5
2.0
Fold Change
GJ_ST
2.0
1.5
VIP
1.0
0.5
0.0
2
1
3
Fold Change
RG_ST
2.0
1.5
VIP
1.0
0.5
UP
Down
Nonsignificant
0.0
1
2
4
6
Fold Change
2.5
DH_ST
2.0
1.5
VIP
1.0
0.5
0.0
2
1
Fold Change
HQ_ST
2.0
1.5
VIP
1.0
0.5
0.0
2
1
3
4
Fold Change
HL_ST
2.0
1.5
VIP
1.0
0.5
0.0
2
3
1
Fold Change
B
C
