# Supplementary material for: Divergent impacts on the gut microbiome and host metabolism induced by traditional Chinese Medicine with Cold or Hot properties in mice
Source: Chin Med. 2022 Dec 26;17:144. doi: 10.1186/s13020-022-00697-2 (PMC9793677; doi:10.1186/s13020-022-00697-2)
Supplement: Supplementary file 4 — Additional file 4. Fig. S4: A Volcano map shows the distribution of metabolites in each group. Red spots presented down-regulated metabolites, while blue spots mean up-regulated metabolites under the condition VIP > 1. B Venn diagram between Hot_LT and Cold_LT based on shared metabolites of each nature. C Venn diagram between Hot_LT and Cold_LT based on shared pathways by metabolic pathway enrichment analysis on KEGG pathway level 3 with condition of pathway impact > 0. D Under enrichment analysis, the pathways satisfying the conditional impact > 0 in each group. [file 13020_2022_697_MOESM4_ESM.pptx]

## Slide 1
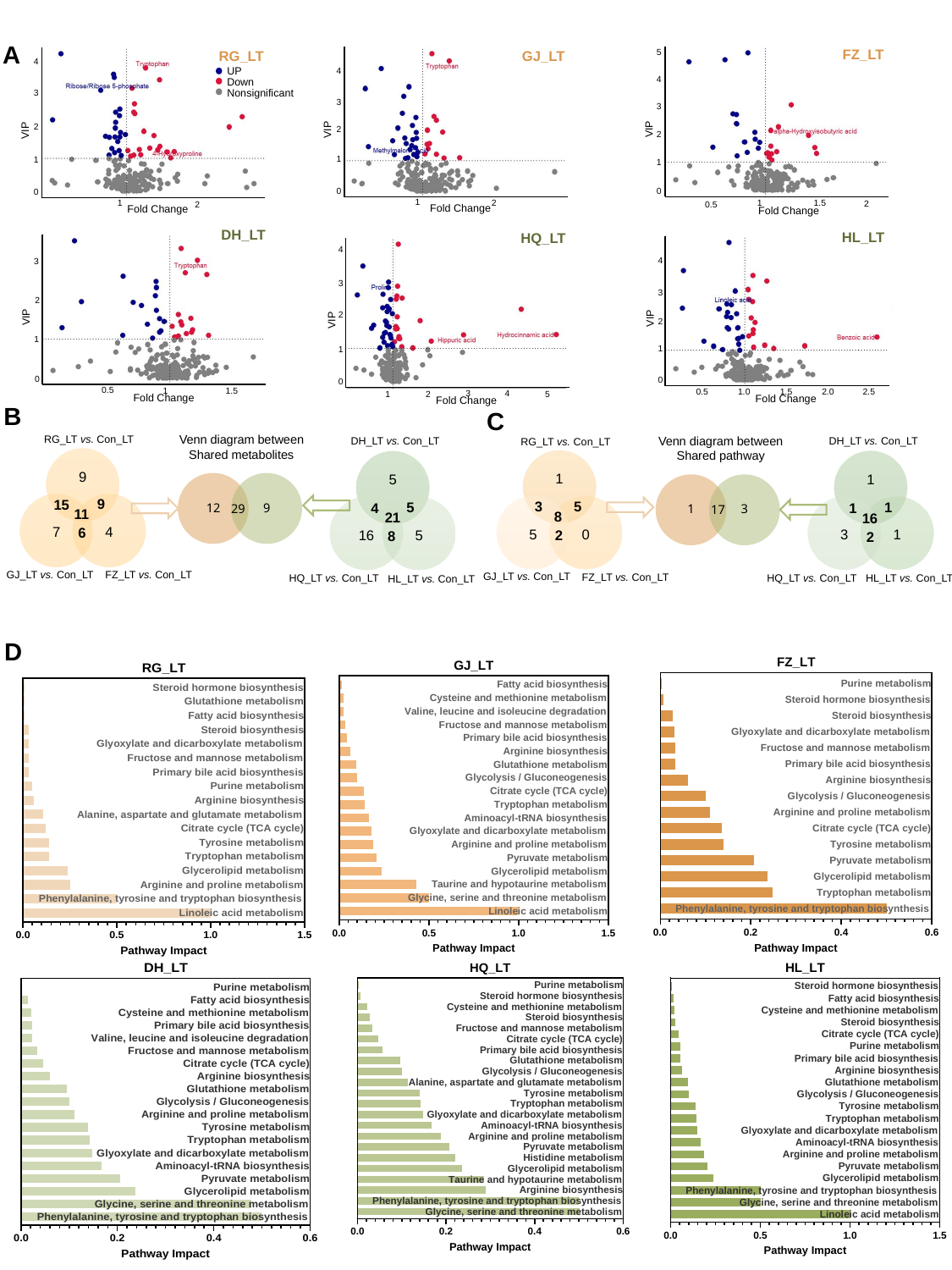

A
5
FZ_LT
4
3
VIP
2
1
0
1.5
1
2
0.5
Fold Change
RG_LT
4
UP
Down
Nonsignificant
3
2
VIP
1
0
1
2
GJ_LT
4
3
VIP
2
1
0
1
2
Fold Change
Fold Change
DH_LT
3
2
VIP
1
0
0.5
1
1.5
Fold Change
HL_LT
4
3
VIP
2
1
0
0.5
1.0
1.5
2.0
2.5
Fold Change
HQ_LT
4
3
2
VIP
1
0
4
3
5
2
1
Fold Change
B
C
Venn diagram between Shared metabolites
RG_LT vs. Con_LT
DH_LT vs. Con_LT
9
15
5
4
29
11
21
6
8
GJ_LT vs. Con_LT
FZ_LT vs. Con_LT
HQ_LT vs. Con_LT
HL_LT vs. Con_LT
Venn diagram between Shared pathway
DH_LT vs. Con_LT
1
1
16
2
HQ_LT vs. Con_LT
HL_LT vs. Con_LT
RG_LT vs. Con_LT
5
3
17
8
2
GJ_LT vs. Con_LT
FZ_LT vs. Con_LT
D
