# Supplementary material for: Divergent impacts on the gut microbiome and host metabolism induced by traditional Chinese Medicine with Cold or Hot properties in mice
Source: Chin Med. 2022 Dec 26;17:144. doi: 10.1186/s13020-022-00697-2 (PMC9793677; doi:10.1186/s13020-022-00697-2)
Supplement: Supplementary file 5 — Additional file 5. Fig. S5: A, B The metabolites involved in specific changed pathways in Hot_ and Cold_TCM. N = 5 each group, Student’s t-test *P < 0.05, **P < 0.01. [file 13020_2022_697_MOESM5_ESM.pptx]

## Slide 1
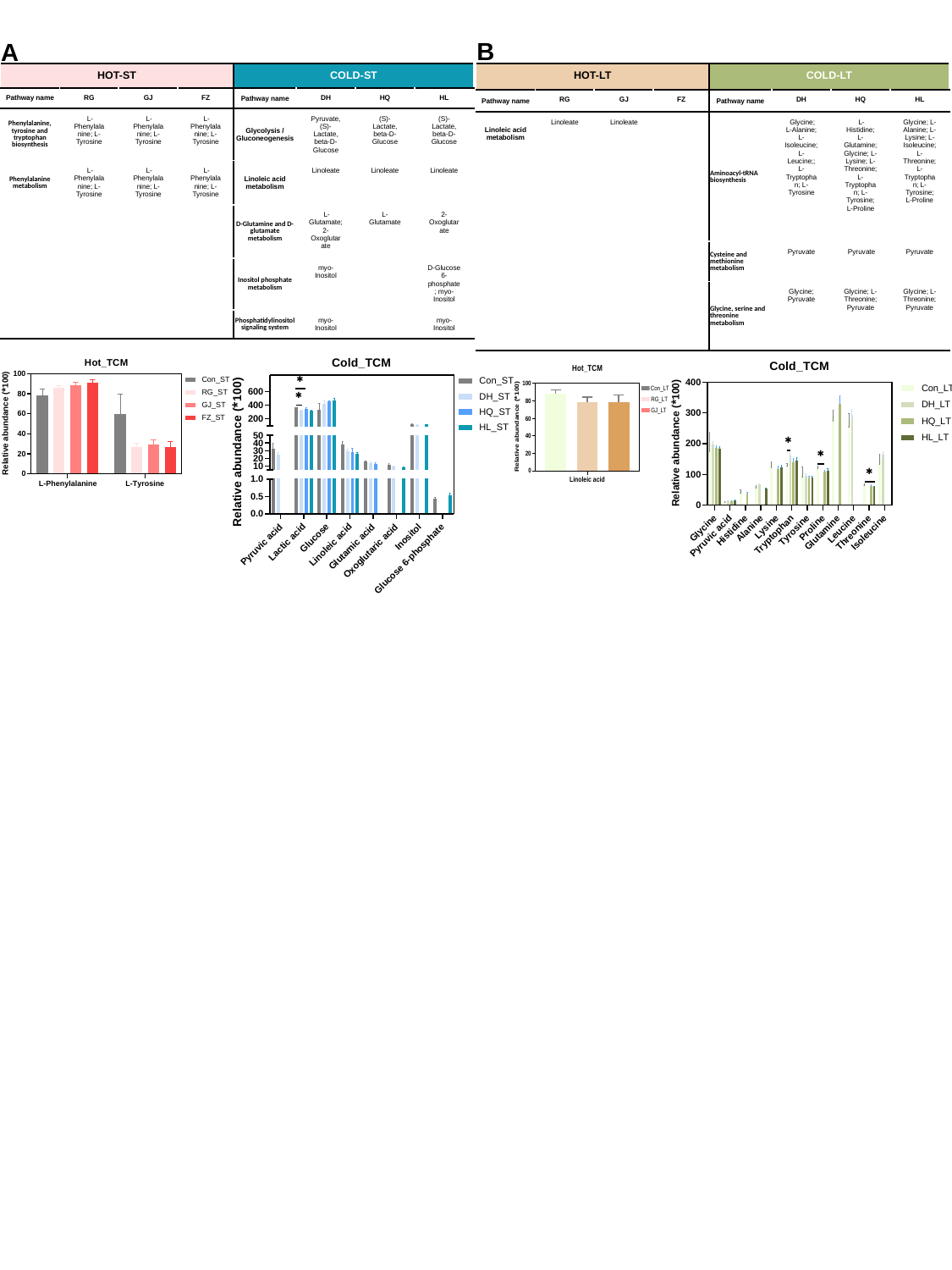

B
A
| HOT-ST | | | | COLD-ST | | | |
| --- | --- | --- | --- | --- | --- | --- | --- |
| Pathway name | RG | GJ | FZ | Pathway name | DH | HQ | HL |
| Phenylalanine, tyrosine and tryptophan biosynthesis | L-Phenylalanine; L-Tyrosine | L-Phenylalanine; L-Tyrosine | L-Phenylalanine; L-Tyrosine | Glycolysis / Gluconeogenesis | Pyruvate, (S)-Lactate, beta-D-Glucose | (S)-Lactate, beta-D-Glucose | (S)-Lactate, beta-D-Glucose |
| Phenylalanine metabolism | L-Phenylalanine; L-Tyrosine | L-Phenylalanine; L-Tyrosine | L-Phenylalanine; L-Tyrosine | Linoleic acid metabolism | Linoleate | Linoleate | Linoleate |
| | | | | D-Glutamine and D-glutamate metabolism | L-Glutamate; 2-Oxoglutarate | L-Glutamate | 2-Oxoglutarate |
| | | | | Inositol phosphate metabolism | myo-Inositol | | D-Glucose 6-phosphate; myo-Inositol |
| | | | | Phosphatidylinositol signaling system | myo-Inositol | | myo-Inositol |
| HOT-LT | | | | COLD-LT | | | |
| --- | --- | --- | --- | --- | --- | --- | --- |
| Pathway name | RG | GJ | FZ | Pathway name | DH | HQ | HL |
| Linoleic acid metabolism | Linoleate | Linoleate | | Aminoacyl-tRNA biosynthesis | Glycine; L-Alanine; L-Isoleucine; L-Leucine;; L-Tryptophan; L-Tyrosine | L-Histidine; L-Glutamine; Glycine; L-Lysine; L-Threonine; L-Tryptophan; L-Tyrosine; L-Proline | Glycine; L-Alanine; L-Lysine; L-Isoleucine; L-Threonine; L-Tryptophan; L-Tyrosine; L-Proline |
| | | | | Cysteine and methionine metabolism | Pyruvate | Pyruvate | Pyruvate |
| | | | | Glycine, serine and threonine metabolism | Glycine; Pyruvate | Glycine; L-Threonine; Pyruvate | Glycine; L-Threonine; Pyruvate |
