# Supplementary material for: Divergent impacts on the gut microbiome and host metabolism induced by traditional Chinese Medicine with Cold or Hot properties in mice
Source: Chin Med. 2022 Dec 26;17:144. doi: 10.1186/s13020-022-00697-2 (PMC9793677; doi:10.1186/s13020-022-00697-2)
Supplement: Supplementary file 6 — Additional file 6. Fig. S6: The main peaks of six extracts. Copitdis Rhizoma was determined by UV chromatograph due to the instable baseline under TOF-MS/MS, others were determined by mass chromatograph. The names and proportions of peaks are shown in Table 1. [file 13020_2022_697_MOESM6_ESM.pptx]

## Slide 1
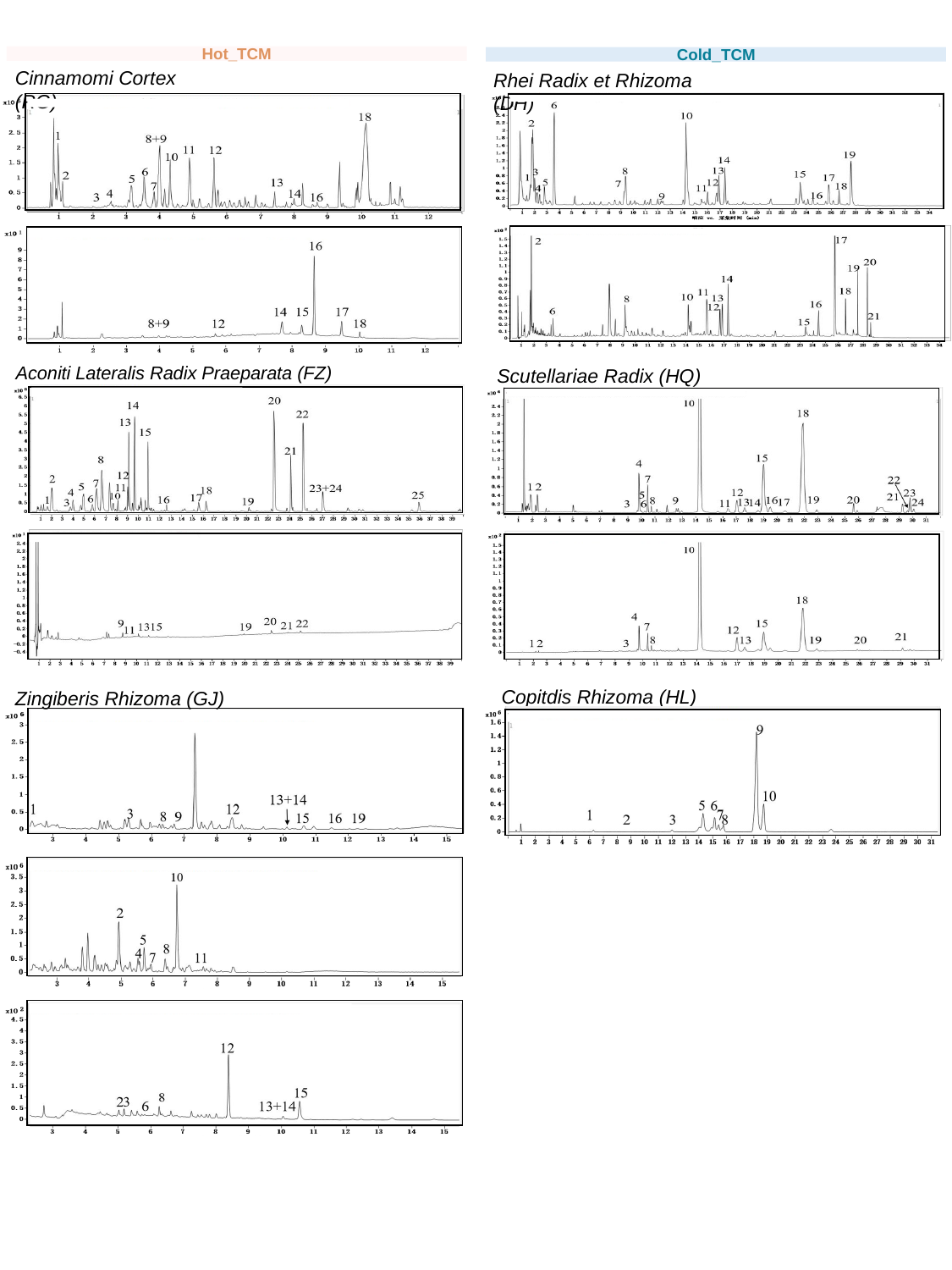

Hot_TCM
Cold_TCM
Cinnamomi Cortex (RG)
Rhei Radix et Rhizoma (DH)
Aconiti Lateralis Radix Praeparata (FZ)
Scutellariae Radix (HQ)
Copitdis Rhizoma (HL)
Zingiberis Rhizoma (GJ)
